# Event-related potentials to repeated speech in 9-month-old infants at risk for autism spectrum disorder

**DOI:** 10.1186/1866-1955-6-43

**Published:** 2014-11-28

**Authors:** Anne Seery, Helen Tager-Flusberg, Charles A Nelson

**Affiliations:** Department of Pediatrics, New York University School of Medicine, New York, NY 10016 USA; Department of Psychological and Brain Sciences, Boston University, Boston, MA 02215 USA; Laboratories of Cognitive Neuroscience, Division of Developmental Medicine, Boston Children’s Hospital, Boston, MA 02215 USA; Harvard Medical School, Boston, MA 02115 USA

**Keywords:** Autism spectrum disorders, Event-related potentials, Speech processing, Infancy, Endophenotype, Auditory evoked potentials, Language

## Abstract

**Background:**

Atypical neural responses to repeated auditory and linguistic stimuli have been reported both in individuals with autism spectrum disorder (ASD) and their first-degree relatives. Recent work suggests that the younger siblings of children with ASD have atypical event-related potentials (ERPs) to repeated tones at 9 months of age; however, the functional significance is unclear, and it is unknown whether this atypicality is also present in response to linguistic stimuli.

**Methods:**

We analyzed ERPs to repetitive and deviant consonant-vowel stimuli at 9 months in 35 unaffected high-risk-for-autism (HRA) infant siblings of children with ASD and 45 low-risk control (LRC) infants. We examined a positive component, the P150, over frontal and central electrode sites and investigated the relationships between this component and later behavior.

**Results:**

Over frontal electrodes, HRA infants had larger-amplitude ERPs to repetitions of the standard than LRC infants, whereas ERPs to the deviant did not differ between HRA and LRC infants. Furthermore, for HRA infants, the amplitude of ERPs to the standards was positively correlated with later language ability.

**Conclusions:**

Our work suggests that atypical ERPs to repeated speech during infancy are a possible endophenotype of ASD but that this atypicality is associated with beneficial, rather than disordered, language development. Potential mechanisms driving these relationships and implications for development are discussed.

## Background

Autism spectrum disorder (ASD) involves social and communicative impairments in addition to restricted interests and repetitive behaviors and is often accompanied by language impairment [1]. Infants with a family history of ASD exhibit subtle atypicalities in how they process and interact with the world during the 1st year of life [2–4], and while these infants have an increased risk for developing ASD and other language or behavioral impairments, the majority are not ultimately clinically impaired [5–7]. A better understanding of how this population processes their environment during infancy and how this relates to their later development can help to understand why these infants vary in developmental outcome. Here, we focus specifically on how infants with a family history of ASD process repeated speech sounds, as atypical neural processing of repeated auditory stimuli have previously been reported both in individuals with ASD and their first-degree relatives [8–13].

Event-related potentials (ERPs) have been used extensively to study auditory and linguistic processing in adults and children with ASD, often by measuring electrophysiological response to a repeated auditory stimulus (a ‘standard’). Individuals with ASD have been shown to exhibit diminished cortical evoked potentials to both linguistic [8–10] and nonlinguistic ([8–13], although see [14]) standard stimuli, which has often been interpreted as reflecting inefficient auditory encoding. In typically developing children, cortical evoked potentials become weaker with successive repetition of an auditory stimulus, and there is evidence that children with ASD do not show this progressive dampening of response [15, 16].

Atypical neural responses to auditory and linguistic stimuli, including responses to repetitive stimuli, are also evident in the unaffected relatives of individuals with ASD [12, 17–19], suggesting that these may serve as familial risk markers of autism; however, these atypicalities can manifest differently in individuals with ASD than in their unaffected family members [12, 17]. For example, children with ASD show dampened evoked potentials to repeated auditory stimuli relative to typical controls, whereas their parents have been reported as having atypically *large* evoked potentials to these stimuli relative to typical adults [12]. No direct comparisons of neural responses to auditory repetition have been made between age- or behavior-matched groups of individuals with ASD and unaffected individuals with a family history of ASD, making interpretation of group differences difficult, and it is possible that group differences in neural response are due to group differences in behavioral characteristics (e.g., cognitive ability and language ability) rather than ASD specifically.

It also remains unclear at what point patterns of atypical auditory and linguistic processing begin developmentally and how this relates to later behavior, both in individuals who develop ASD and relatives who do not. ASD is highly heritable, and approximately 20% of infants with an older sibling with ASD (i.e., high-risk-for-autism infants, or HRA infants) will develop the disorder themselves [20]. In these infants who develop ASD, overt behavioral symptoms begin to emerge by around 12 months of age and are often sufficiently pronounced and stable enough to support a diagnosis by 24 months of age [2, 3, 21–23]. A substantial portion of the remaining HRA infants who do *not* develop ASD begin to display subclinical autism-like behavioral traits (e.g., subthreshold ASD symptoms or subtle delays in language) indicative of the broader autism phenotype (BAP) by around 12 months of age [5]. Furthermore, before 12 months, HRA infants as a group exhibit subtle behavioral and neurological atypicalities in how they process and interact with the world [24–30], potentially reflecting intermediate traits (‘endophenotypes’) of ASD [31, 32].

Subtle atypicalities related to behavioral and neurological processing of auditory and linguistic stimuli have been reported over the 1st year of life in HRA infants [26, 33–36]. However, very few studies have examined responses to repeated auditory or linguistic stimuli, and only one has looked *specifically* at how the brain responds to auditory repetition. In that study, Guiraud et al. [26] used an ‘oddball paradigm’ to present 9-month-old infants with a repeated standard tone interspersed with infrequently presented deviant stimuli and then analyzed how a positive ERP component, the P150, changed in response to consecutive presentations of the standard. For low-risk control (LRC) infants, the amplitude of the P150 became progressively smaller after each presentation of the standard, reflecting neural habituation to the repetitive stimulus, while HRA infants in contrast failed to exhibit this neural habituation. That study, though, did not examine relationships between altered ERPs to auditory repetition and behavior, so the functional implications of this are currently unknown.

Even less is known about how HRA infants process linguistic repetition. Seery et al. [35] examined this to some degree using an oddball paradigm similar to Guiraud et al. [26] but with consonant-vowel speech stimuli rather than tones. Few group differences were evident between HRA and LRC infants in their P150s to the standard stimuli (although group differences in lateralization of a later ERP component were evident across condition types); however, this study only included standard stimuli that were immediately followed by a deviant (i.e., ‘pre-deviant’ standards) rather than examining consecutive presentations of the standard. It is possible then that HRA infants in that study *did* have altered responses to repetitions of the standard stimulus, similar to what has been reported for tones [26], but that this was not captured due to examining only pre-deviant trials.

In the current study, we explored the possibility that HRA infants have atypical ERPs to repetitive speech stimuli, potentially similar to what has been reported for tones [26], by focusing on infants’ P150s to consecutively presented standards in a sample expanded from Seery et al. [35]. In addition to examining whether ERPs to repetitive speech are altered in HRA infants, we sought to further understand the functional significance of any such atypicality by examining the relationships between ERPs to repetitive speech and infants’ later behavioral characteristics. Finally, to determine whether any such atypicality is specific to the repetitive nature of the stimulus or whether it would occur in response to speech in general, we also examined responses to the deviant stimulus, although our previous work suggested that HRA infants do not differ from low-risk infants in their P150s to deviant speech sounds.

## Methods

The work presented here was part of a larger longitudinal investigation of infants at risk for ASD. Infants were recruited into the larger study between birth and 6 months and participated in a battery of behavioral, electrophysiological, and eye-tracking tasks during laboratory visits at several different ages (3, 6, 9, 12, 18, 24, and 36 months). The focus here is on portions of the electrophysiological data collected during the 9-month visit. All work was approved by the Institutional Review Boards at Boston Children’s Hospital and Boston University, and upon enrollment, parents provided written consent for their infant to participate in the longitudinal study.

### Participants

Two groups of infants (HRA and LRC) from monolingual, English-speaking households (English spoken ≥75% of the time) were enrolled. HRA infants each had an older sibling with ASD. Diagnoses in the older siblings were provided by expert community clinicians and were not due to known genetic disorders (e.g., fragile X syndrome), as determined by a detailed screening interview. LRC infants had at least one typically developing older sibling and no known first-degree relatives with ASD or other neurodevelopmental disorders, based on the screening interview. Infants were excluded if they had a gestational age less than 36 weeks, a genetic disorder known to be related to ASD, extensive perinatal/postnatal medical or neurological problems, or exposure to any language that uses the paradigm’s nonnative phonemic contrast (e.g., Hindi or Bengali; see the ‘Stimuli and procedure’ section for more details).

Usable ERP data were obtained from 85 9-month-old infants: 40 from HRA (mean age in days (SD) = 280.7(10.2); 21 male) and 45 from LRC (mean age in days (SD) = 281.3(10.8); 23 male). An additional 69 infants were tested but were not included in analyses due to the following: a) refusal to wear the ERP net, becoming too fussy after an initial visual ERP task, or not completing the task (7 HRA, 11 LRC); b) not providing enough artifact-free data due to excessive movement/fussiness or having excessively noisy data after editing (21 HRA, 26 LRC); c) experimenter/equipment error (1 HRA); d) English spoken in the house less than 75% of the time (2 HRA); or e) exposure to Hindi (1 HRA).

Because HRA infants who develop ASD differ in many ways from HRA infants who do not, and in order to better focus on the majority of infants who do not develop ASD, we excluded any infants with known ASD diagnoses from further analyses. Diagnoses were made using the Autism Diagnostic Observation Schedule (ADOS) [37], administered at 24 and 36 months alongside expert clinical judgment at 36 months. The ADOS, a semi-structured play-based interaction designed to measure autism symptoms, provides a ‘severity score’ (1–10) to capture the presence of ASD symptoms, with higher scores indicating greater symptom severity and scores of 4 or higher being indicative of ASD. Infants were classified as having ASD if they scored at or above the ASD cutoff of 4 on their most recent 24- or 36-month ADOS and, for participants with 36-month outcome data available, received a clinical judgment rating of ‘ASD.’ Five HRA infants received diagnoses of ASD using these criteria (four from the ADOS at 36 months, one from the ADOS at 24 months) and were excluded from analyses. One LRC infant scored at the ASD cutoff on the ADOS at his 36-month visit; however, he scored below the threshold at 24 months and received a clinical judgment of ‘typically developing’ at 36 months, so he remained in the sample. Nine HRA infants and 10 LRC infants had not yet completed an ADOS at either 24 or 36 months and were allowed to remain in analyses.

Thirty-five HRA and 45 LRC infants were included in the final sample. Behavioral characteristics of the participants near the time of ERP collection (9 months) were obtained using the Mullen Scales of Early Learning [38], a developmental assessment, at 6 and 12 months. From the Mullen, we obtained standardized *T*-scores for four subscales: Fine Motor, Visual Reception, Expressive Language, and Receptive Language. In line with other work on this population, we found no difference between groups (using independent-samples *t*-tests) on subscale *T*-scores at 6 months (all *p* > 0.5) but found that HRA infants scored significantly lower than LRC infants on the Visual Reception and Receptive Language subscales at 12 months (all *p* < .05). Additional details about behavioral and demographic information for these groups are provided in Table 1. Note that 46 of these infants (23 HRA, 23 LRC) were included in Seery et al. [35].

**Table 1:** **Characteristics of participants included in analyses**

	Group		
	HRA (SD)	LRC (SD)	***p*** value
*N*	35	45	
Male: female	18:17	23:22	
Race (% nonwhite)	2.9	13.6	
Ethnicity (% Hispanic or Latino)	8.6	2.3	
Family income level (% less than $75,000)	16.7	15.2	
Maternal education (% less than college degree)	32.3	8.3	
Geodesic sensor net:Hydrocel sensor net	17:18	18:27	
NetAmp200:NetAmp300	25:10	32:13	
S1 trials	26.8 (7.6)	26.4 (7.4)	
S2 trials	22.2 (5.9)	21.2 (6.5)	
S3 trials	18.3 (5.2)	16.8 (4.6)	
Deviant trials	26.1 (7.1)	25.3 (7.0)	
6-month Mullen *T*-scores (*31 HRA*, *37 LRC*)			
Visual reception	49.69 (9.0)	47.86 (7.3)	*.378*
Fine motor	48.38 (7.4)	47.84 (6.9)	*.765*
Receptive language	49.54 (8.4)	47.65 (5.7)	*.293*
Expressive language	45.85 (6.1)	46.51 (5.8)	*.662*
12-month Mullen *T*-scores (*38 HRA*, *41 LRC*)			
Visual reception	53.03 (8.2)	57.20 (8.4)	.036*
Fine motor	58.73 (10.7)	61.98 (8.3)	*.146*
Receptive language	42.94 (10.5)	46.83 (7.4)	*.067*
Expressive language	46.15 (12.4)	52.12 (8.1)	.015*
18-month Mullen *T*-scores (*33 HRA*, *35 LRC*)			
Visual reception	48.33 (7.4)	52.47 (7.9)	.035*
Fine motor	51.97 (7.0)	53.60 (6.0)	*.313*
Receptive language	43.40 (13.3)	55.71 (12.8)	<.001**
Expressive language	47.97 (9.5)	52.89 (7.0)	.019*
18-month ADOS (*31 HRA*, *35 LRC*)			
Severity score	2.66 (1.9)	1.54 (0.9)	.002**

Note that not all families provided demographic information.

*p < .05, **p < .01.

Behavioral characteristics of the infants were further assessed using the Mullen and ADOS at 18 months. Previous work has shown that at this age, traits of the broader autism phenotype (such as elevated ASD symptoms and language delay) are clearly evident, yet ASD symptoms are still not stable enough to allow for firm ASD diagnoses [5, 21]. As expected, at 18 months, HRA infants performed significantly lower than LRC infants on the Visual Reception (*p* = .035), Receptive Language (*p* < .001), and Expressive Language (*p* = .019) subscales of the Mullen and had significantly higher ADOS severity scores than LRC infants (*p* = .002). See Table 1 for more details as well as the number of infants who provided Mullen and ADOS scores at each age.

### Stimuli and procedure

A stream of consonant-vowel stimuli was presented to infants using a double oddball paradigm. A standard stimulus (voiced, unaspirated, retroflex stop; /ɖa/) was presented 80% of the time, and the primary deviant stimulus (voiceless, aspirated retroflex palatal stop; /ta/) was randomly interspersed 10% of the time. A second, nonnative language deviant (voiced, unaspirated dental stop; /da/) was presented the remaining 10% of the time. English does not differentiate between the voiced retroflex and dental stops, so adult monolingual English speakers perceive both the standard and the nonnative deviant simply as /da/. In contrast, these sounds can be easily distinguished by adult speakers of languages that use these sounds contrastively (e.g., Bengali or Hindi) and by very young infants being exposed to any language.

A maximum of 600 stimuli, each 300 ms in duration, were presented at 80 db over two bilateral speakers using an interstimulus interval with offset-to-onset times varying between 1,100 and 1,400 ms. Throughout the procedure, infants were seated on a parent’s lap in a sound-attenuated, dimly lit room and wore either a 64-channel Geodesic Sensor Net or 128-channel HydroCel Geodesic Sensor Net (Electrical Geodesics Inc., Eugene, OR; change in net type was due to a system upgrade partway through the longitudinal project) from which we recorded continuous electroencephalogram (EEG). To maintain infants’ interest and increase toleration of the electrode net, an experimenter was present and provided the infant with opportunities for quiet toy play, bubble blowing, feeding, or other similar activities. On average, the procedure took approximately 15 min. More detailed information about stimulus creation and procedure has been provided previously in Seery et al. [35].

### Analysis of electrophysiological data

Continuous EEG was referenced online to vertex (Cz), amplified with a 0.1-to-100-Hz band-pass filter using a NetAmp200 or NetAmp300 amplifier (due to a system upgrade partway through the longitudinal project), and digitized at 250H using NetStation software (Electrical Geodesics Inc.). EEG was segmented into 800-ms epochs starting 100 ms before stimulus onset, digitally filtered using a 30-Hz low-pass elliptical filter and baseline-corrected using mean voltage during the 100-ms pre-stimulus baseline period.

Segments were visually examined for artifacts, and individual channels were marked as bad if contaminated by artifacts such as body movement, eye movement, eye-blinks, or off-scale activity (±200 μV). If more than 15% of the channels in a given segment were marked as bad, that entire segment was excluded from analyses. Participants with fewer than ten acceptable segments in any stimulus category (see below) were excluded from all analyses. For the remaining participants, the bad channels of accepted segments were replaced using spherical spline interpolation, then average waveforms for each condition were calculated and re-referenced to the average reference.

For the current study, we were specifically interested in how infants’ brains respond to repetitions of the standard stimulus. Note that infants’ ability to detect nonnative phonemic contrasts changes drastically over the 1st year of life, thus impacting their ERPs to the nonnative deviant stimulus [39, 40]. Examination of this developmental change is outside the scope of the current project, so responses to the nonnative deviant were not examined here (although the reader is directed to Seery et al. [35]). Instead, analyses were restricted to the native deviant (hereafter referred to simply as the deviant) as well as ‘runs’ of consecutively presented instances of the standard stimulus. Following Guiraud et al. [26], we segmented the continuous EEG into four categories of stimuli. The first category consisted of the deviant stimulus (**/ta/**), and the remaining three categories were constructed from the runs of repeated standards that began immediately after this deviant (e.g., /da/ /ta/ **/da/ /da/ /da/**). All standards that *immediately* followed a native deviant were included in the ‘first standard’ category (S_1_; /ta/ **/da/**). The ‘second standard’ (S_2_) category included all standards that immediately followed an S_1_ (i.e., the two stimuli presented just before the S_2_ were the deviant and then an S_1_; /ta/ /da/ **/da/**). Finally, the ‘third standard’ (S_3_) category included all standards that immediately followed an S_2_ (/ta/ /da/ /da/ **/da/)**.

It should be noted that since the paradigm was designed such that deviant stimuli were randomly interspersed throughout the procedure, there were fewer instances of S_2_ stimuli than S_1_ and fewer S_3_ than S_2_ (due to the deviant stimuli ‘interrupting’ the runs of consecutive standard stimuli). The number of usable trials per condition directly contributes to the signal-to-noise ratio of ERPs, and the signal-to-noise ratio can have an impact on both the maximum amplitude and latency of ERPs [41]. Therefore, we analyzed average amplitude of the waveform rather than maximum amplitude or latency to the peak, since average amplitude is less impacted by differences in number of trials [41]. The number of usable trials did not differ across groups for any of the four conditions (all *p* > .05; see Table 1).

**Figure 1:**
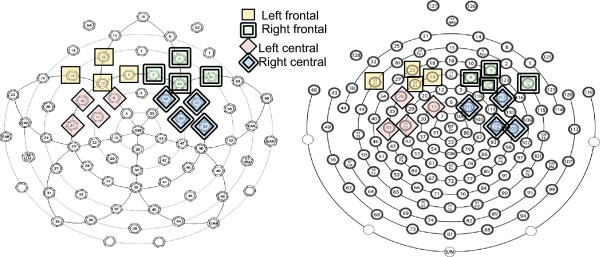
**Regions of interest.** Electrode groupings used for the 64-channel Geodesic Sensor Net (on left) and the 128-channel HydroCel Sensor Net (on right). The frontal regions of interest (ROIs) consisted of electrodes F1, F3, F7, and AF3 on the left and F2, F4, F8, and AF4 on the right. Central regions of interest consisted of FC1, FC5, C3, and C5 on the left and FC2, FC6, C4, and C6 on the right.

Based on previous work, we focused analyses on an early positive component, the P150, which is sensitive to stimulus deviance and is maximal over frontal and central electrodes [26, 35, 42]. Specifically, we analyzed the average amplitude of the waveform from 150–300-ms post stimulus onset over four regions of interest (ROIs) computed from frontal and central electrodes from the left and right hemisphere (see Figure 1 for details).

## Results

Following Guiraud et al. [26], and in line with the focus of this study, we focused primarily on responses to the repetition of the standard stimulus. Specifically, we examined whether infants differ in how they respond to the first, second, and third consecutive repetitions of this stimuli and whether HRA and LRC infants differ from each other. We next examined the relationships between these responses at 9 months and later behavior at 18 months. Finally, in order to understand whether any potential atypicalities are related specifically to the *repetition* of the stimulus or whether they are more generally related to speech processing, we examined infants’ responses to the deviant stimulus.

Mixed-model ANOVAs were used, with Greenhouse-Geisser corrections applied as needed, and significant effects were examined further using reduced ANOVAs, independent-sample *t*-tests, or paired-sample *t*-tests, with Bonferroni corrections applied when appropriate. An alpha level of .05 was used throughout.

**Figure 2:**
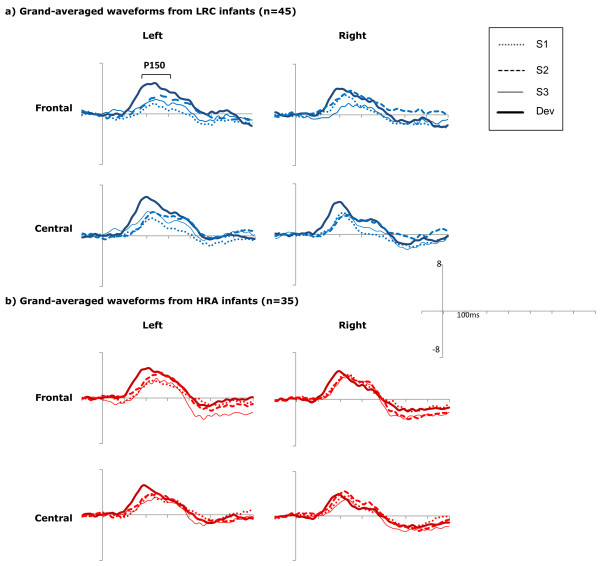
**Grand-averaged waveforms for LRC and HRA infants.** Grand-averaged waveform graphs over each ROI for **(a)** LRC and **(b)** HRA groups for the first (S1), second (S2), and third (S3) standards as well as the deviant (Dev) condition.

Waveform graphs for the three standard stimuli as well as for the deviant are given in Figure 2.

### How do infants respond to repeated speech?

To examine how infants respond to repetitions of the standard, we performed separate mixed-model ANOVAs for frontal and central ROIs using condition (first standard, second standard, third standard) and hemisphere (left, right) as repeated factors and group (HRA, LRC) as a between-subjects factor with the average amplitude of the P150 as the dependent variable.

**Figure 3:**
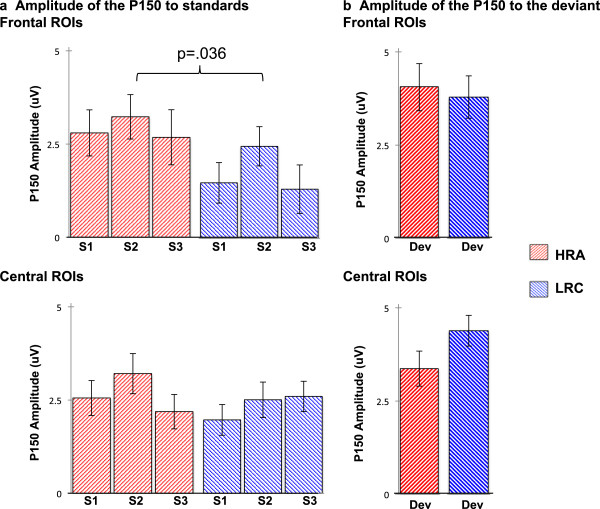
**Amplitude of the P150 to standard and deviant stimuli.** Amplitude of P150 for HRA (*red*) and LRC (*blue*) infants. **(a)** Response to the three standard conditions (S1 = first standard, S2 = second standard, S3 = third standard); **(b)** response to the deviant.

Over frontal electrodes, this three-way ANOVA revealed no significant effects of condition but did reveal a main effect of group (F(1,78) = 4.57, *p* = .036; partial eta squared = .055) such that average amplitudes were larger (more positive) for HRA infants (mean = 2.90 μV, SD = 2.54) than LRC infants (mean = 1.76 μV, SD = 2.24; see Figure 3a). There were no significant effects or interactions over central electrodes (all *p* > .20).

### What is the relationship between infants’ ERPs to repeated speech and later behavior?

Next, we investigated the functional implications of elevated ERPs to the standards in HRA infants by exploring the relationship with later behavioral traits at 18 months. As there were no differences in response to the first, second, or third repetitions of the standard in either group or across hemisphere, we averaged the ERPs across these three conditions and across hemispheres. As noted earlier, at 18 months HRA infants had lower Visual Reception, Expressive Language, and Receptive Language *T*-scores as well as higher ADOS severity scores than LRC infants (see Table 1).

We were interested in and anticipated potentially different relationships between ERPs and behavior for LRC versus HRA infants, so we analyzed each group separately, computing bivariate Pearson correlations between the average response to the standards at 9 months and Mullen verbal (Expressive Language, Receptive Language) and nonverbal ability (Visual Reception, Fine Motor) at 18 months. For the HRA infants, we also examined the relationship with autism symptoms by computing nonparametric Spearman correlations between ERPs and ADOS severity scores at 18 months (LRC infants did not have enough variance in severity scores to allow for meaningful correlations).

**Figure 4:**
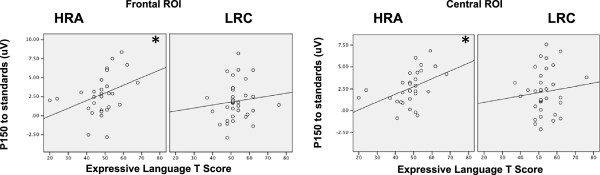
**Correlations between P150 to the standards at 9 months and Expressive Language at 18 months.** Scatterplots of the relationships between amplitude of the P150 to the standards at 9 months and Expressive Language *T*-scores at 18 months for HRA and LRC infants over frontal and central ROIs. Correlation coefficients are given in Table 2. Note that correlations remain significant when excluding the two HRA participants with lowest language scores.*p ≤ .05

For LRC infants, there were no significant correlations between ERPs and any of the behavioral variables. In contrast, for HRA infants, there were positive correlations between amplitude of the P150 to the standards and Expressive Language score over both frontal (Pearson’s *r* = .360, *p* = .051) and central ROIs (Pearson’s *r* = .442, *p* = .015). A summary of correlation coefficients is given in Table 2, and scatter plots for the relationships between amplitude of the P150 and Expressive Language are given in Figure 4.

**Table 2:** **Correlation coefficients between amplitude of P150 to the standards at 9 months by ROI and behavioral variables at 18 months** ^**a**^

	LRC				HRA				
	Receptive language	Expressive language	Visual reception	Fine motor	Receptive language	Expressive language	Visual reception	Fine motor	ADOS severity
*ROI*									
Frontal	-0.038	0.110	-0.064	-0.205	0.185	0.360*	0.160	-0.115	0.140
Central	0.129	0.106	0.154	-0.314	0.147	0.442*	0.190	-0.052	0.278

*p < .05.

^a^Spearman’s rho is given for correlations with ADOS severity score; Pearson’s *r* is given for all other correlations.

### How do infants respond to deviant speech?

We next examined responses to the deviant by computing mixed-model ANOVAs for frontal and central ROIs using hemisphere (left, right) as a repeated factor and group (HRA, LRC) as a between-subjects factor with average amplitude of the P150 to the deviant as the dependent variable.

Over frontal electrodes, this revealed no significant main effects or interactions. Over central electrodes, the ANOVA revealed a main effect of hemisphere (F(1,83) = 7.03, *p* = .010; partial eta squared = .083) such that amplitude was larger over the left hemisphere (mean = 4.64 μV, SD = 3.61) than right (mean = 3.27 μV, SD = 3.63), but no effect of group, suggesting that the response to the deviant did not differ between HRA and LRC infants (Figure 3b).

Finally, we computed an omnibus ANOVA including standards and deviants with condition (first standard, second standard, third standard, deviant) and hemisphere (left, right) as repeated factors and group (HRA, LRC) as a between-subjects factor.

Over frontal electrodes, this ANOVA revealed a main effect of condition (F(3,234) = 4.50, *p* = .005; partial eta squared = .054) modulated by a condition by hemisphere interaction (F(3,234) = 3.14, *p* = .027, partial eta squared = .039). The condition by hemisphere interaction was driven by the fact that within the left hemisphere, the deviant was significantly larger than the first (*p* < .001) and third standards (*p* = .013), while in the right hemisphere there were no differences between conditions (all *p* > .1). Furthermore, the first standard was larger over the right than the left hemisphere (*p* = .014). There were no other significant effects or interactions.

Over the central ROI, this ANOVA revealed only a main effect of condition (F(3,234) = 5.62, *p* = .001; partial eta squared = .067), such that response to the deviant was larger than to the first (*p* = .001) and third standards (*p* = .013), although it did not differ from the second standard (*p* = .136). Response to the three standards did not differ from each other (all *p* > .2).

## Discussion

In this study, we examined how unaffected 9-month-old infants at high risk for ASD respond to repeated speech sounds. Analyses focused on the average amplitude of a positive ERP component, the P150, over frontal and central electrodes in response to three consecutive repetitions of a standard consonant-vowel stimulus. Overall, we found that for frontal electrodes, the amplitude of the P150 to repetitions of the standard was larger for HRA infants than for LRC infants. Furthermore, for HRA infants only, amplitude of ERPs to the standards was positively related to later language ability.

First, consider the LRC infants, whose P150s to the standard did not change in amplitude in response to successive presentations of the standard. In previous work with infants of the same age but using nonlinguistic stimuli, Guiraud et al. [26] reported that the P150 dampened with each consecutive presentation of a repetitive tone, suggesting neural habituation. The difference in these findings may be due in part to the linguistic nature of our stimuli. It is known that typically developing infants prefer to listen to speech over nonspeech sounds and have larger P150s in response to speech sounds than nonspeech sounds [43, 44]. Furthermore, although localization of the neural sources of infant ERP components is challenging, previous work suggests that the infant auditory P150 has generators in the anterior cingulate cortex (ACC) as well as the left and right auditory temporal cortices [42]. When the P150 is elicited by speech sounds, there is evidence that the ACC, which is involved in modulation of attention, is activated before the auditory cortex [42]. The opposite activation pattern (activation of the auditory cortex followed by the ACC) has been found for nonspeech sounds [45], suggesting that attention may play a vital role in the generation of the P150 when infants encounter linguistic sounds. It is possible as well that evidence for neural habituation would be found if we had examined more than three consecutive presentations of the standard (for example, including out to the fourth or fifth standard); however, this was not possible with our data due to a relatively small number of fourth and fifth standard trials. Despite the lack of dampening of the P150 to the repeated syllable, it was clear that infants in our sample were sensitive to the repeated nature of the stimulus, as they showed a strong mismatch response to the deviant as evidenced by larger amplitude to the deviant than the first and third standards.

Next, consider the HRA infants, who, like the LRC infants, had consistent amplitude of the P150 in response to the three consecutive presentations of the standard, although this amplitude was larger than that of LRC infants. These atypically large responses to the standard stimuli provide evidence that processing of repetitive speech sounds by unaffected HRA infants is altered relative to the typical population. This finding builds upon previous work surrounding nonlinguistic auditory repetition processing in infant and adult participants with a family history of ASD [12, 26] by providing evidence that this extends to the linguistic domain during infancy. Importantly, atypically large ERPs to the repetitive speech sounds in HRA infants were associated with *better* development, so atypically elevated responses in our sample appear to be indicative of beneficial rather than impaired processing, particularly as this relates to language acquisition.

There are a few potential explanations for why HRA infants in our study had atypically elevated responses to the repeated speech sounds and why this was associated with better language development. One possibility is that HRA infants experience atypical attention modulation and integration that affects their ERPs to the stimuli during this passive task. There is evidence that HRA infants and older first-degree relatives of individuals with ASD have atypical attention styles [29, 46–48], lending support to this hypothesis. In this scenario, elevated responses to the standards could arise from HRA infants actively attending or listening to the ‘background’ repetitive speech stimuli, in contrast to the LRC infants who may be better at simultaneously attending to multiple features in their environment (e.g., bubbles, toys, food, or other sounds that are present during the task in addition to the background syllables). Previous work suggests that there is variation among infants in their relative preference for listening to speech over other nonlinguistic auditory stimuli and that, as a group, HRA infants show dampened preference for speech over nonspeech sounds relative to LRC infants [36]. However, those HRA infants who *do* prefer to listen to speech have better language ability and later show fewer ASD symptoms [36]. Our findings, then, may be driven by the subset of HRA infants who prefer to attend to speech over nonspeech sounds as these infants may have elevated P150s (due to attending to the speech stimuli) as well as higher later-language ability. If this hypothesis is true, then we would not necessarily expect to find the same functional significance for atypical responses to repetitive *non*linguistic stimuli. Specifically, if response is governed by attention to the stimuli, then previously reported dampened habituation and/or atypically large responses to repeated nonlinguistic sounds in high-risk relatives may be associated with language or sensory integration difficulties. In line with this idea, recent work suggests that children with ASD with auditory hypersensitivity have atypically large neural responses to repeated tones, while children without auditory hypersensitivity do not [49]. Much more work is required in order to understand whether this is the case in unaffected family members as well.

Another potential explanation for the elevated amplitudes to repeated speech sounds in HRA infants is that this arises not from differences in attention but from altered processing of speech more generally. For example, it may be that HRA infants experience difficulties with neural auditory or linguistic processing and that the HRA infants who develop better language also allocate more, or potentially alternative, cognitive or neural resources to processing these stimuli, resulting in larger-amplitude ERPs to the standard. Recent work suggests that brain areas associated with language processing, including the temporal cortex, have atypicalities from early in life in individuals with ASD [50], which could result in impaired auditory and linguistic processing in ASD; however, this has not yet been studied during the 1st year of life in HRA infants.

Future work is needed to elucidate the nature of our findings; however, these data at least provide support for the idea that atypical neural processing of auditory repetition may be an endophenotype of ASD. Notably, HRA infants did not differ from LRC infants in their responses to the deviant, suggesting that this endophenotype may be specific to the processing of repetitive stimuli. Although we did not find a significant group-by-condition interaction between how HRA and LRC infants processed standard and deviant stimuli, providing some hesitation to this interpretation, previous work also suggests no differences between HRA and LRC infants in P150s to deviant speech stimuli [35]. More work should continue to explore this issue.

Our work has some limitations that should be considered when interpreting the findings. First, we examined only responses to linguistic stimuli, so it is unclear whether our findings are specific to linguistic stimuli or whether they hold also for nonlinguistic auditory stimuli. As discussed earlier, future work should directly address this empirical question. Second, we did not examine infants who develop ASD, so it is unclear whether similar responses would also be found in HRA infants who *do* ultimately receive diagnoses of ASD. Comparison of infants who develop ASD against HRA infants who do not may additionally help to understand previously reported differences in neural responses to auditory/linguistic stimuli between older children with ASD and their first-degree relatives. It should also be noted that it is possible that some HRA infants from our sample may ultimately develop ASD as not all infants had reached an age where diagnosis is possible.

## Conclusions

In sum, we found evidence that atypically large ERPs to repeated speech sounds are present in unaffected 9-month-old HRA infants and may be an endophenotype of ASD. Large responses were associated with better developmental outcome, suggesting that this response pattern, although atypical, is not indicative of disordered processing. Although more work is needed in order to understand these findings, this adds to a growing body of literature suggesting that HRA infants differ from low-risk infants in how they process and interact with their auditory world.

## Abbreviations

ACC: anterior cingulate cortex
ADOS: Autism Diagnostic Observation Schedule
ASD: autism spectrum disorder
BAP: broader autism phenotype
EEG: electroencephalogram
ERP: event-related potential
HRA: high-risk for autism
LRC: low-risk control
ROI: region of interest.
